# Food Waste to Livestock Feed: Prospects and Challenges for Swine Farming in Peri-urban Sri Lanka

**DOI:** 10.1007/s43615-022-00168-8

**Published:** 2022-04-12

**Authors:** Nilanthi Jayathilake, Mohamed Aheeyar, Pay Drechsel

**Affiliations:** International Water Management Institute (IWMI), Battaramulla, 10120 Sri Lanka

**Keywords:** Food security, Circular economy, Reuse, Piggeries, Urban food waste, Regulations

## Abstract

Using farm animals for their natural capability of “recycling” food waste (FW) that is unfit for direct human consumption can support a circular economy as shown in the case of Sri Lanka’s Western Province. The reuse of organic residues including FW as animal feed is a traditional agricultural practice in Sri Lanka but is less studied within an urban FW context. A survey of piggeries using FW in and around the rapidly urbanizing city of Colombo showed that FW is a major feed source in the farms accounting for on average 82% of total feed. About 40% of the farms collected the FW mainly from hotels, restaurants, and institutional canteens. Urban FW is supplied to farmers free of charge when collected directly from the sources, although 26% of the farmers collected FW via intermediaries against a fee. As FW is collected daily, the restaurants appreciate the reliable service, the farmers the low-cost feed, and the municipality the reduced FW volumes to be collected. However, this triple-win situation encounters challenges such as (tourist related) seasonal low supply, which was exacerbated under the Covid-19 lockdown of food services. Another area of concern refers to biosafety. Although the large majority of interviewed farmers boil FW which contains raw meat or fish, there is a paucity of related guidelines and control. Given the benefits of FW use, it is worthwhile to explore how far these informal partnerships could be scaled without increasing transport costs for farmers, while introducing biosafety monitoring. For now, the regulatory environment is highly siloed and does not support material transitions across sector boundaries towards a circular economy.

## Introduction

Globally one-third of food produced, approximately 1.3 billion tonnes, is wasted [1]. Reducing food loss and waste (FLW) essentially plays a key role in achieving global food security. It is estimated that by using less than a quarter of the food wasted, the approximate global population of 1 billion hungry people could be freed from malnutrition [2]. On the other hand, the agricultural food system is globally one of the largest single contributors of greenhouse gases (GHG), a significant source of pollution of land, water sources, and oceans, and depletes non-renewable resources [3]. Reducing food waste (FW) prevents the waste of land, water, energy, and other resources embedded in food and is therefore not only essential for improving the sustainability of food systems but also for generating a win for the economy, for food security, and the environment [4]. These key factors provide strong motivations for reducing FW. Estimation of energy return to investment for the current food production system indicates the requirement of more energy input than its output indicating the unsustainability of the system [5].

Reducing FLW has become an even greater global priority with the commitments to achieve Sustainable Development Goal (SDG) 12.3, which calls to halve per capita FLW by 2030. It is also an important target in achieving other SDG targets relating to food and nutrition security, environmental sustainability, and so forth [6, 7].

Numerous interventions have been launched worldwide at the supranational level, national level, and at community level in terms of guidelines and pledges for FW reduction. The “food waste hierarchy” promoted, e.g., by USEPA and FAO aims to, firstly, support the prevention of FW; secondly, to facilitate the distribution of food that is still edible but not marketable; thirdly, for what remains, use as animal feed; and fourthly, use as compost and/or energy and use disposal in landfills as the least preferred option [8].

According to recent estimates, about 7000 tonnes of solid waste (SW) are generated in Sri Lanka per day, of which about 4000 tonnes (57%) is FW [9, 10]. Although treatment of organic waste (in general) via composting has been on the national agenda for several years, strategies for FW prevention, reduction, and reuse along the food chain have only very recently been promoted in the country [11]. Compared with rural areas where organic residues and FW are commonly re-integrated into the local agricultural production cycle, the mountain of FW generated in urban areas puts significant pressure on municipal waste management and the urban environment. The amount of wasted food stands in stark contrast to the nutritional deficits observed especially in many urban households [12, 13] compared with rural ones with a higher consumption of animal-sourced foods [14]. Transforming FW into meat, eggs, and milk could reduce the loss of valuable nutrients.

### Reusing Urban FW in Swine Farming in Sri Lanka

Leveraging farm animals for their natural capability of feeding on and digesting a wide variety of organic matter, including FW, that is unfit for direct human consumption is at the core of an enhanced circular agro-food system where livestock act as efficient bio-processors to generate edible products high in protein with lower environmental and health impacts compared to processing waste through composting or anaerobic digestion [15–18].

Historically, using FW as animal feed has been a common practice in household-level piggeries and the practice has been adopted in commercial-scale animal farming. As commercial animal feed is the largest single cost item for livestock production, accounting for 60 to 85% of the total cost of production [19], low-cost FW is a preferred alternative or supplement [20]. Feeding practices of pig production systems, especially medium- and large-scale farms, target a mix of swill (food scrap) feeding and concentrate feeding [21]. According to Bandara et al. (2014) [22], swill feeding was practiced by 92% of pig farmers in Ratnapura District of Sri Lanka, but most of them (65%) were not using it for breeding stock. The high demand for alternative feed sources is reflected in the disconnection between the increasing number of pigs in Sri Lanka between 2015 and 2019 (+ 72%), while swine feed production declined over the same period (− 37%) [23].

**Fig. 1:**
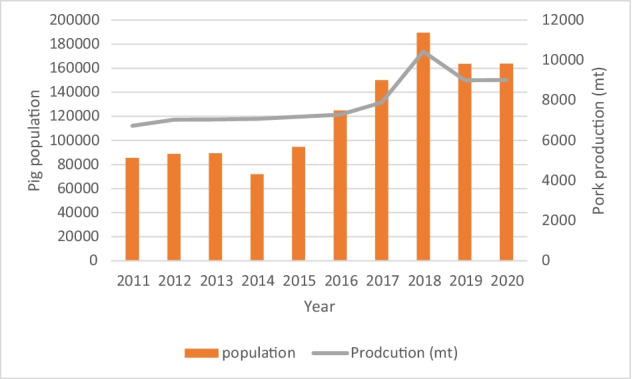
Pig population and pork production in Sri Lanka (2011–2020). Data source: Department of Animal Production and Health, Sri Lanka^1^

Swine farming is one of the main livestock sub-sectors in the country and serves as the primary source of income for 5000 to 7000 farmers [23, 24]. It is on the increase (Fig. 1) and considered a highly profitable livestock sector due to the simple management and feeding practices involved and so far, low disease occurrence [25]. Pig farming is most popular in the rapidly urbanizing Western Province (Colombo, Gampaha, and Kalutara) and the adjacent Puttalam district, together traditionally known as the “pig belt” [25]. About 50% of total pig production occurs in this area while the Western Province of the country constitutes one-third of the total national pig population [23].

### FW Potential in Sri Lanka with Special Reference to the Western Province

In Sri Lanka, the food service sector has been informally diverting FW to piggeries as a strategy for FW management for many years [26, 27]. The Western Province with the commercial capital of Sri Lanka, Colombo, generates the highest amount of solid waste in the country amounting to 33% of the total SW which consequently represents the highest FW generation within Sri Lanka [9, 28]. The three major SW disposal sites in the Western Province—Kaduwela, Karadiyana, and Kerawalapitiya—receive a waste load of 1,317 tonnes per day from 20 local authorities in the province, of which 724 tonnes are estimated to be FW [9].

The quantity of FW generated in the municipalities ranges from 50 to 69% of the total SW (Table 1). For example, the estimated amount of FW generated in Colombo Municipal Council (CMC) area was estimated as 353 tonnes/day in 2017, which is half of the total waste generated in this geographical area. Waste analysis conducted for segregated waste collected by the CMC shows that it has primarily consisted of household FW, followed by 110 tonnes/day from food services, 25 tonnes/day from markets, and 9 tonnes/day from slaughterhouses and meat shops [29].

**Table 1:** Estimated amount of FW in the SW generated in selected highly populated municipalities in Sri Lanka

Municipal council	Population (2022)	Total SW quantity (tonnes/day)	FW quantities (tonnes/day)	FW percentage	References
Colombo	648,034	706	353	50%	[29]
Moratuwa	185,031	125	65	52%	[28]
Jaffna	169,102	105	72	69%	[28]
Kandy	111,701	127	74	58%	[28, 30, 31]
Batticaloa	86,742	53	30	57%	[32, 33]
Kurunegala	28,571	48	25	52%	[28]

Source: [9], population estimates from https://worldpopulationreview.com/countries/cities/sri-lanka

With its international airport and capital city, the Western Province also has the highest number of hotels at the provincial level in Sri Lanka. Major hotels in the CMC and suburbs, institutional canteens, and hospitals have been key sources of FW for piggeries. Related data are however not captured in any waste statistics except a few ad hoc studies. For example, Sandaruwani and Gnanapala (2016) [34] found that 79% of the solid waste generated by the hotels in Colombo was FW.

This paper aims to understand the use of urban FW as feed in piggeries in urban vicinity, and the opportunities and challenges of this practice. The study also focuses on understanding the level of engagement of the key players in the industry and how effective this practice can be as an FW reduction strategy, in particular to the urban food service sector, with due attention to existing legislations [35] and possible food safety challenges.

## Methodology

### Sample and Questionnaire

For this study, sample farmers were purposefully selected from the registered pig farmers’ list provided by the National Livestock Development Board (NLDB) targeting farmers in the Western Region known by extension officers for their likely interest in FW. Given the informality of the practice, and lack of related data, those farmers were inquired if they know other farmers in the area using FW (snowballing), resulting eventually in a sample of 24 farmers for the survey (4.8% of all registered swine farmers in the Western Province). Most of the sample piggeries were scattered in Kaduwela, Welivita, Hanwella, Kosgama (Colombo District), Ja-Ela (Gampaha District), and Maggona (Kalutara District). Given the Covid-19 restrictions in the country, the telephone survey method was adopted during May 2020 to collect necessary data. A semi-structured questionnaire was developed that comprised sections corresponding to farm characteristics, FW sourcing (including details on collected amounts, sources, collection frequency, and traveling distance), feed utilization, cost data, and challenges encountered by the food supply chain, including the situation in crisis in the food supply chain (i.e., due to the Covid-19 pandemic).

### Data Analysis

Data gathered during the telephone interviews were incorporated into an Excel sheet under each question. The responses received were essentially satisfactory; however, there was a challenge in processing the financial information such as cost of operation and profits, due to reporting discrepancies and a resulting low level of confidence in the data. Reasons might include reluctance to reveal financial data [over the telephone] or unavailability of proper data records. Poor record-keeping by swine farmers was also reported by Bandara et al. [22]. Hence, the financial data were not processed to produce results in this study. The study also estimated the potential FW absorption capacity by the pig sector based on official data on the pig population in the Western Province.

### Additional Data

Data on FW generation were recorded during 7-day audits in several urban sectors in 2020 in an accompanying joint project led by FAO. A summary of the data will be presented while details are reported in three forthcoming (2022) FAO, IWMI reports (see references). The audits did not aim to be representative and were affected by Covid-19 restrictions. The main aim of the exercise was awareness creation and methodology testing.

## Results and Discussion

### Scale of the Piggery Farms

**Fig. 2:**
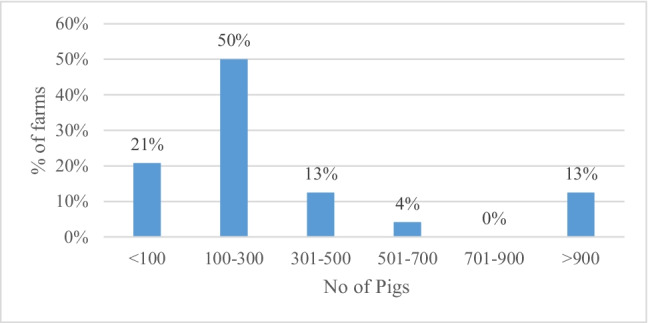
Number of pigs in surveyed piggeries. Source: Authors’ survey data in 2020

Half of the sampled farms (50%) were rearing 100 to 300 pigs while the total sample had an animal distribution of 10–5000 (Fig. 2). In terms of scale, most farms (62%) can be classified as large scale in line with swine farming classification in Sri Lanka (Table 2). Kothalawala et al. (2008) [21] reported that, in Sri Lanka, about 60% of the farms were small scale (< 50 animals), 25% were medium scale (51–100 animals), and 15% were large scales. According to this classification, 67% of the sample captured in this study represents large-scale farms, whereas 21% and 13% represent medium- and small-scale farms, respectively. Hence, the findings from this study are more applicable to large-scale farms.

**Table 2:** Classification of sample swine farms (based on the number of adult animals per farm)

Scale	Number of farms
Domestic scale (1–4 adult animals)	1
Small scale (5–25 adult animals)	7
Medium scale (26–49 adult animals)	1
Large scale (> 50 adult animals)	15

Source: Authors’ survey data in 2020; classification scale by [36]

Results revealed that in the interviewed sample, FW constitutes on average 82% of the total feed requirement. The proportion of FW to conventional feed may vary based on the purpose and the type of pigs raised on their farms,^2^ the composition, and the quality of FW. Kothalawala et al. (2008) [21] reported, for example, that under large-scale swine farming in Sri Lanka, a mixed byproduct feeding system was more profitable compared to swill feeding or concentrate feeding.

### Feed Sources and Collection

Feed supply was reportedly collected mostly from entities located in the CMC followed by the Kaduwela area. This could be attributed to the highest percentage of food service entities located in the commercial capital to cater to the large working force. About 26% of the farmers collected FW from hotels and restaurants only, while another 26% were mainly collecting from institutional canteens. However, a larger percentage (39%) collected the feed from multiple sources including hotels, hospitals, and institutional canteens to meet their demand. Around 42% of the farmers collected the FW from less than five collection points, while 38% collected from 5 to 10 collection points and 17% from more than 15 collection points daily. Informal communication networks for sourcing FW were reported by 62% of farmers.

**Fig. 3:**
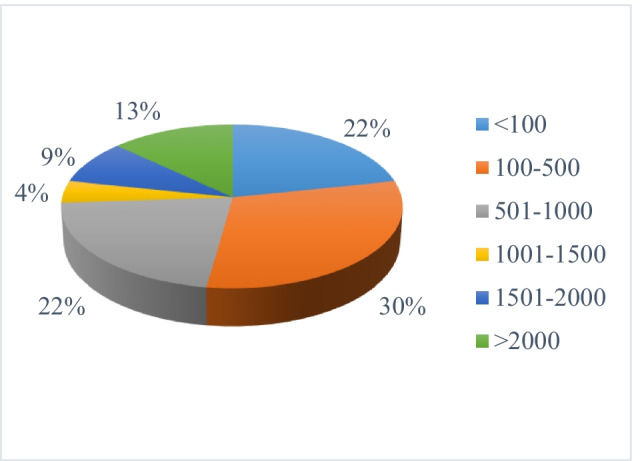
Average amount of FW collected by pig farmers (kg/day). Source: Authors’ survey data in 2020

The average amount of FW collected per day by piggery farmers varied with factors such as the size of the piggery and the availability of feed. The survey indicated a significant variation between 50 and 10,000 kg of feed collection among the farmers with the majority (75%) collecting less than 1,000 kg of FW per day (Fig. 3). In terms of composition, the majority (70% of the farmers) collected FW in the form of a mix of many food commodities while 30% mainly collected rice and cooked FW.

### Operational Aspects: Transport and Cost

It was noted that 96% of the pig farmers collected fresh feed daily. Farmers travel on average 38 km back and forth to collect the feed. This is because the piggeries are mostly located in the peri-urban areas in the Colombo, Gampaha, and Kalutara districts and the feed supply comes mostly from the larger business entities located in urban Colombo. Although the distance appears to be considerable, it implies that the value of (low cost) FW as a feed has been well recognized by the farmers. However, given the bias of the sample to larger farms, it should not be concluded that also smaller farms can afford the transport.

Minimizing the number of collection points while maximizing the collected FW volumes per point is important from a transport cost perspective, and to keep the FW as fresh as possible. In an ideal scenario, just one major supplier would fill the truck. Although there are many sectors which provide a high percentage of FW, of which 46–70% is at the moment of disposal still edible for humans (Table 3), the largest overall share derives from households but with the smallest volume per collection point. In these cases, it would help farmers to access possible solid waste transfer stations, compost plants, or landfills with separate organic waste processing.

**Table 3:** Results of 7-day audits of food waste generation in Colombo in 2020

Type of case study	Origin of FW	Method of assessment	Key findings
Food service	Hotel	A 7-day FW audit was conducted in one top-end hotel located in Colombo (with several banquet halls and restaurants able for catering to over 4,000 guests in peak periods)	The hotel generated 2.4 tonnes of FW during the week of which more than 2/3 was edible at the time of discard Edible FW consisted of 88% of cooked rice, starchy foods, vegetables, and fruits and 12% of fish and meat
	Restaurant	A 7-day FW audit was conducted at 5 selected restaurants in a food court in Colombo	The selected restaurants generated 126 kg of FW in the week out of which about 70% was edible. The edible FW mainly consisted of rice (47% of total FW) and vegetables (22% of total FW)
Retail and wholesale market	Wholesale market	A 7-day FW audit was conducted at two vegetable shops, two fruit shops, and two meat shops in the market in Colombo	740 kg of FW generated in the selected fruit and vegetable shops in the week. About 46% of the FW was edible
	Retail market	A 7-day FW audit was done at two vegetable stalls, two fruit stalls, one fish stall, one meat stall, and one dry fish stall located in the retail market in Colombo	The FW generated in the stalls amounted to 2 tonnes during the week About 80% of the FW was vegetables and fruit and 90% of the vegetable waste was edible
	Vegetable and fruit retail stall	A 7-day FW audit was conducted at a retail stall in Colombo	The stall generated 327 kg of FW during the week. About 65–85% of FW generated in the stall was edible
	Supermarket	A 7-day FW audit was conducted at an outlet of a popular supermarket chain in Colombo	Total FW generated in the week was 445 kg. Vegetable waste was highest (48%) followed by fruits (24%) and cooked rice and starchy products (25%) About 84% of the FW was edible
Caterers	Hospital	A 7-day FW audit was conducted in two selected hospital wards and the hospital kitchen	FW generated in the selected two wards, hospital kitchen and staff dining area was 493 kg in the week. About 65% of the FW was edible
Households	Middle-income households	A 7-day FW audit was conducted in 5 middle- income households in Colombo	Total FW generated by the 5 households was 137 kg; 41% of FW was edible with 60% of edible waste being rice
	Low-income households	A 7-day FW audit was conducted in 5 low-income households in Colombo	Total FW generated by the 5 households was 92 kg; 50% of FW was edible with 55% of edible waste being rice

Source: [37]

The use of FW as feed lowers pig production costs due to the low cost of FW compared to conventional feeds. It was reported in the European Union (EU) that feed costs ranged from 55 to 72% of total pig production costs [38]. Research conducted in Bihar, India, shows that the feed cost was up to 72 to 80% of the total cost [39]. According to data available, the practice of utilizing FW as feed by Sri Lankan pig farmers is a rational decision, given that feed cost accounted for about 68% of the total cost of production of pork [22]. On the other hand, this is a win–win situation also for the food service sector as the waste is diverted reliably, daily, and at no cost.

While most farmers received the feed supply free of charge, 26% of the farmers paid a price ranging from LKR 2–40/kg with more than 50% paying LKR 10/kg when the waste was supplied through intermediaries.^3^ This practice was also reported from the neighboring Negombo Municipal Council where approximately 1 to 2 tonnes of FW was collected by private traders to sell as animal feed for piggery farmers [31]. FW generated in Kandy and Peradeniya Teaching Hospitals located in the Central Province of the country was also sold to piggeries [27]. This indicates the possibility of creating business opportunities for private sector engagement that can consequently expand the sector to create more revenue and jobs. In countries such as Japan and South Korea, businesses have been established to collect and process FW and sell it to farmers [40].

### FW for Animal feed? Current Challenges

Given that the pig farmers mostly receive FW free of charge, they use it as the major feed source instead of commercial concentrated feed. However, the seasonal nature of the supply of FW linked with tourism and festive seasons and inadequate supply of feed under the Covid-19 pandemic (and lockdown of restaurants) are key challenges. Feed shortage under Covid-19 resulted in increased cost of operations for sourcing commercial feed. Farmers indicated a 50–60% income loss due to the unavailability of the FW during the peak of the pandemic.

A more technical challenge related to FW is that it must be fully segregated from other waste to avoid nonfood materials such as plastic, polythene, or glass, entering the feed. It is important to collect FW separately from other waste and in a sufficiently fresh condition as it is being practiced in several SE Asian countries. In South Korea and Taiwan, for example, FW can constitute 81 and 72% of animal feed, respectively [41]. According to the current legislation of SW management in Sri Lanka, source segregation is expected from a policy perspective, but its enforcement remains a challenge, and especially at the household level non-satisfactory [42]. Separating organic and inorganic parts at farm would result in additional [labor] costs for pig farmers in case they start targeting FW collected from households.

FW may also potentially pose a biosafety risk, as meat residuals can transmit Food and Mouth Disease (FMD) and African swine fever unless the waste is processed [18]. For example, in the EU, the use of catering waste in animal feed was prohibited in 2002 after the FMD outbreak that took place in 2001, due to the feeding of uncooked FW to swine [38, 43, 44]. Utilization of FW as animal feed essentially requires adequate pre-treatment and adherence to a clear guideline for storage and way of feeding. Proper heat treatment would be sufficient to inactivate harmful pathogens and the safety for treated FW has been demonstrated in many studies [15, 44]. In countries such as Japan and South Korea where FW is commonly used as animal feed, the usage has been closely regulated through legislation to ensure proper heat treatment, appropriate storage, and transport of FW [44]. Countries such as Malaysia have introduced policy instruments as a control measure to tackle such challenges; for example, the Feed Act (Act 698) (45) stipulates that not all FW is suitable for use as animal feed for livestock except for the more common practice of harvesting of carcass bones and eggshells [41].

Although safety and hygiene of the feed have been a concern at the farmers’ end from the business perspective and at the authorities’ end from a health perspective, currently no national regulations are guiding the safe use of FW as animal feed in Sri Lanka, while there are many technical options [38]. Despite this lack of regulations, most of the farmers (88%) stated that they boil the collected FW (containing raw meat, fish, or poultry waste) before feeding it to their animals, but not already cooked dishes. Answers varied, however, e.g., in view of the boiling duration (45 min to 4–5 h), or the guidance farmers use, with most farmers confirming the lack of any.

Foot and mouth disease is common in Sri Lanka, although so far mostly in cattle and buffaloes. It usually coincides with the seasonal movement of livestock returning to the villages as a part of extensive and uncontrolled livestock management practice in Sri Lanka’s dry zone. Swine were affected by the Porcine Reproductive and Respiratory Syndrome (PRRS) with over 200 deaths in 2020, mostly in the Central and North Central provinces. Feeding of untreated swill and poor or no biosecurity practices in swine farms were named as the main sources of the virus [24].

### Potential of FW as Animal Feed

Based on the data gathered during the survey, it was calculated that in total, 34 tonnes of FW from the Colombo area were absorbed daily by the surveyed piggeries. However, the amount collected by the farmers varied between 50 and 10,000 kg/day depending on demand and supply, with 75% of farmers collecting less than 1,000 kg/day. On average, large-scale farmers collect about 2,060 kg/day, whereas medium-scale and small-scale farmers collect about 255 kg/day and 60 kg/day, respectively. Swill feed per pig per day was estimated to be on average 3.4 kg/day.

According to the 2019 database from the Department of Animal Production and Health, Sri Lanka, there are 503 registered pig farms^4^ in the Western Province with 53,225 animals [23]. If all the pig farmers would (e.g., for financial reasons) favor FW as feed, the sector could absorb as much as 181 tonnes FW per day, which is about 20% of what the authorities collect and with few exceptions dump in landfills [10, 42]. Although these estimates need to be verified, given the sampling bias to larger farms, the high number of piggeries within the Western Province shows the potential of the sector to reduce the FW volume and contribute to the circular economy, while helping to reduce the carbon footprint of the swine sector through reduced commercial feed production/use, and avoided GHG emission from landfills [40].

## Conclusions

The informal partnerships between peri-urban piggeries and the food service sector in Sri Lanka’s commercial capital Colombo represent a success story of a circular economy business model [46] which is based on a triple win situation, where (i) larger piggeries save on commercial feed by collecting unconsumed food from the food service sector, which would otherwise end on landfills; (ii) the municipal waste management department has a lower FW volume to transport and manage; and (iii) the urban food service sector benefits from a reliable daily waste collection, which is free of charge, and reduces the sector’s dependency on the often-irregular municipal waste collection and requirement of paying (an informal or formal) collection fee.

However, the “up-cycling” of FW to livestock feed is not without challenges. These concern two main areas:

- Lack of a regulatory environment and institutional support: Regulations are needed especially to observe feed safety and feed quality in view of FMD, but also other hazards, such as non-infectious pathogens, or physical and chemical contaminants, as they can occur in particular in urban areas [47]. Capacity development and institutional support could help to facilitate formal arrangements between FW supply and demand, and the safe processing of FW on farm. However, the implications of formalizing the existing (well-functioning but informal) model need to be well-assessed prior to implementation.
- Year-round FW supply: Seasonal variations in FW supply are a challenge where restaurants and hotels depend on tourists. Expanding the collection area will increase transport costs and can be difficult for smaller swine enterprises. The supply gap could be closed through FW generated by other sources, but is constrained in view of households by the limited number of (or access to) organic waste transfer stations, landfills, and resource centers, and the limited quality of domestic waste segregation.

So far, FW prevention and reduction are not directly included in policies and regulations, and only the least preferred alternative of the “food waste hierarchy,” i.e., composting, is supported to minimize the waste volume going to landfill sites [35]. However, the promotion of source segregation is weak, and without policy support, local authorities have not yet developed by-laws or guidelines for the food service sector related to FW prevention, reduction, reuse, and recycling. At the other “end” of the circular value chain, the existing legislations related to animal feed or feed ingredients in Sri Lanka, such as the Animal Feed Act No.15 of 1986/2016, solely aim at commercial feed [48] and do not consider alternative sources, such as “free” food waste. Thus, the current regulatory environment is highly siloed and does not anticipate and support material transitions across sector boundaries towards a circular economy.

To overcome these challenges, awareness creation along the food value chain as well as at the policy level is required. In 2019, FAO initiated such a dialogue in Sri Lanka which resulted in 2021 in a National Roadmap on Urban Food Waste Prevention and Reduction for households, food services, retailers, and wholesalers [49]. A central part of the awareness creation was to demonstrate the financial dimensions of FW to business entities [50].

How to reconcile the biosafety issues is of particular importance. In addition to the direct transactions between the waste suppliers and the farmers, feed supply is sometimes carried out through intermediaries. These different operating models need to be further explored to find viable but also safe business models for the benefit of all stakeholders. Multiple health risk barriers will likely be needed as well as related options for compliance monitoring. Although most pig farmers confirmed that they boil FW which contains raw meat or fish, actual practices vary and need to be standardized. Farmers’ responses show risk awareness which will support the promotion of hygienic practices, but also requires key actors such as local veterinary and health authorities to monitor safe reuse. Countries such as the UK have stipulated policies on animal feed, which can be referred to in formulating contextualized guidelines. This process must be supported by institutional capacity development given the lack of a proper monitoring system in the feed industry in general, and inadequate laboratory facilities for quality assurance [48].

The presented biosafety challenges are common across South Asia and relatively better addressed in South-East Asia with a higher pig density, governmental attention to the sector, and vaccination rates. Pig systems in both regions are in a transformation from rural backyard to intensive commercial system, especially in peri-urban areas. The pace of growth is faster in South-East Asia than in South Asia, which provides an opportunity for cross-regional learning [51]. This applies in particular to Japan, Taiwan, South Korea, and Thailand which have proactively supported the benefits of recycling FW into animal feed by developing appropriate biosafety regulations and infrastructure to accomplish this. Moreover, to support a conversion at scale, South Korea banned the disposal of FW in landfills [52].

## Footnotes

1 http://www.daph.gov.lk/web/images/content_image/Livestock_stat/key_stat/2021/swine2020.pdf

2 Most of fattening farms used food waste as their main feed source while breeder farms used more concentrated feed.

3 USD 1.00 = LKR 190 in May 2020.

4 The Department of Census and Statistics lists 1,174 farmers raising swine in 2019 which might also include those where pigs are a minority aside other livestock. http://www.statistics.gov.lk/Agriculture/StaticalInformation/rubb7
